# Copper *bis*-Dipyridoquinoxaline Is a Potent DNA Intercalator that Induces Superoxide-Mediated Cleavage via the Minor Groove

**DOI:** 10.3390/molecules24234301

**Published:** 2019-11-26

**Authors:** Zara Molphy, Vickie McKee, Andrew Kellett

**Affiliations:** 1 School of Chemical Sciences, National Institute for Cellular Biotechnology and Nano Research Facility, Dublin City University, Glasnevin, Dublin 9, Ireland; zara.molphy@dcu.ie (Z.M.); vickie.mckee@dcu.ie (V.M.); 2 Synthesis and Solid-State Pharmaceutical Centre, School of Chemical Sciences, Dublin City University, Glasnevin, Dublin 9, Ireland; 3 Department of Physics, Chemistry and Pharmacy, University of Southern Denmark, Campusvej 55, 5230 Odense M, Denmark

**Keywords:** DNA damage, copper, chemical nuclease, intercalation, free radical oxidation

## Abstract

Herein, we report the synthesis, characterisation, X-ray crystallography, and oxidative DNA binding interactions of the copper artificial metallo-nuclease [Cu(DPQ)_2_(NO_3_)](NO_3_), where DPQ = dipyrido[3,2-*f*:2′,3′-*h*]quinoxaline. The cation [Cu(DPQ)_2_]^2+^ (Cu-DPQ), is a high-affinity binder of duplex DNA and presents an intercalative profile in topoisomerase unwinding and viscosity experiments. Artificial metallo-nuclease activity occurs in the absence of exogenous reductant but is greatly enhanced by the presence of the reductant Na-*L*-ascorbate. Mechanistically, oxidative DNA damage occurs in the minor groove, is mediated aerobically by the Cu(I) complex and is dependent on both superoxide and hydroxyl radical generation. To corroborate cleavage at the minor groove, DNA oxidation of a cytosine–guanine (5′-CCGG-3′)-rich oligomer was examined in tandem with a 5-methylcytosine (5′-C5mCGG-3′) derivative where 5mC served to sterically block the major groove and direct damage to the minor groove. Overall, both the DNA binding affinity and cleavage mechanism of Cu-DPQ depart from Sigman’s reagent [Cu(1,10-phenanthroline)_2_]^2+^; however, both complexes are potent oxidants of the minor groove.

## 1. Introduction

**Figure 1:**
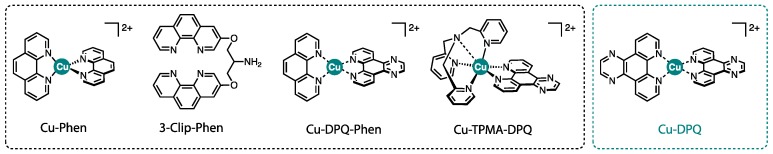
Structures of Cu-Phen, 3-Clip-Phen ligand and ternary copper(II) complexes incorporating 1,10-phenanthroline (Phen) and dipyridoquinoxaline (DPQ) Cu-DPQ-Phen and Cu-TPMA-DPQ developed in our laboratory [32]. The current study focuses on the preparation and chemical nuclease activity of Cu-DPQ (assuming dissociation of nitrato ligand in solution).

Since the structural elucidation of duplex DNA, the construction of small molecules that recognise and react at specific sites to modify DNA structure, reactivity and biological repair processes has been an area of considerable research interest. The discovery of the synthetic chemical nuclease [Cu(1,10-phenanthroline)_2_]^2+^ (Cu-Phen) in 1979, sparked efforts toward the development of new artificial metallonucleases with DNA cleavage mediated through the generation of reactive oxygen species (ROS) at the DNA interface [1]. A kinetic study revealed that the nuclease activity of the Cu(II) complex firstly involves reduction to the activated Cu(I) species, which binds reversibly to double-stranded DNA, while the second step involves oxidation of the non-covalently bound Cu(I) species by H_2_O_2_ (or O_2_) to generate reactive oxygen species (ROS) responsible for strand scission [2]. Cu-Phen degrades DNA in a mechanism involving hydrogen atom abstraction from the C1′ deoxyribose position, resulting in the production of the C1′ radical as a major oxidative product. The C1′ site is only accessible by minor groove binding agents oriented toward H-1′, such as Cu-Phen and its derivatives [3,4]. Other targets of oxidative attack via the minor groove include C-H bonds located at C4′ and C5′ positions that are well characterised sites of attack by Mn(TMPyP)/KHSO_5_, iron bleomycin and the enediyne antibiotics [5,6,7,8,9,10]. Cu-Phen and several derivatives have shown promising antitumoral [11,12], antifungal [13], antimicrobial [14,15] and protein engineering properties [16]. However, limitations associated with the Cu-Phen scaffold include (*i*) weak DNA binding constant; (*ii*) binds both DNA and protein biomolecules without specificity; (*iii*) a high dissociation constant of the second coordinated 1,10 phenanthroline ligand; and (*iv*) a reliance on an exogenous reductant (ascorbate or thiols) and oxidant to initiate ROS production to mediate C–H bond activation and strand scission [7]. Accordingly, modulation of the Cu-Phen scaffold represents an interesting developmental challenge. To circumvent dissociation of the second Phen group, the Clip-Phen ligand was developed to link both Phen ligands via their C2- or C3-carbons by a short flexible arm (Figure 1) [17,18]. Another significant property of this ligand is that its primary amine can be functionalised with sequence-directing groups including minor groove binders netropsin and distamycin [19,20,21,22,23], intercalating acridine conjugates [24], or oligonucleotides [25] to localise nuclease activity.

In recent years, we have focused on introducing modifications to the Cu-Phen scaffold to enhance DNA recognition, oxidative damage and cytotoxicity [11]. Approaches including inner sphere modifications using coordinated carboxylate groups [13]; dicarboxylate groups (e.g., o-phthalate or octanedioate) [26,27,28,29,30,31]; designer phenazine intercalators [32,33]; tris-(2-pyridylmethyl)amine (TPMA) caging ligands [34,35]; and the introduction of a binuclear *di*-cation have been undertaken [26,30,36]. Previously, we reported a series of phenazine-functionalized Cu(II) phenanthroline complexes—with general formula [Cu(N,N′)(Phen)]^2+^ (where N,N′ = dipyridoquinoxaline (DPQ) or dipyridophenazine (DPPZ))—that offered a significant enhancement of DNA binding affinity relative to Cu–Phen [32]. A microfluidic “on-chip”-based method identified Cu-DPQ-Phen as the most active chemical nuclease in the series and later, the hydroxyl radical was identified as the predominant species responsible for the oxidative cleavage event [33]. We also probed the chemical nuclease efficacy on restricted plasmids with varying GC content. Significant differences were noted in the chemical nuclease efficacy of the Cu-N,N′-Phen complex series compared to Cu-Phen. Cu-Phen displayed higher chemical nuclease activity on pUC19 (51% GC), while each of the Cu-N,N′-Phen complexes had enhanced activity on the pBC4 plasmid (59% GC), leading us to hypothesise that this general class of compound may oxidatively target cytosine–phosphate–guanine (CpG) islands.

Our interest in developing new chemical nucleases with high efficacy and enhanced targeting properties prompted this study, where we report the synthesis and X-ray structural characterisation of [Cu(dipyrido[3,2-*f*:2′,3′-*h*]quinoxaline)_2_(NO_3_)](NO_3_). The aim of this study was to investigate what influence two DPQ ligands have within the core Cu-(N,N′)_2_ complex when applied as an artificial chemical nuclease. Additionally, the DNA binding and oxidative DNA damage profile of Cu–DPQ was compared to the well-studied Cu-Phen and Cu–DPQ-Phen complexes previously developed in our laboratory [32,33].

## 2. Experimental

### 2.1. Materials and Methods

Chemicals and reagents were sourced from Sigma-Aldrich (St. Louis, MO, USA) or Tokyo Chemical Industry (TCI, Oxford, UK Ltd.) and were used without further purification. High-performance liquid chromatography (HPLC)-grade chloroform (CHCl_3_), methanol (MeOH) and acetonitrile (CH_3_CN) were used as received. ^1^H and ^13^C-NMR spectra were obtained using a Bruker AC 400 and 600 MHz NMR spectrometer. pH was monitored by a Mettler Toledo InLab Expert Pro-ISM pH probe (Columbus, OH, USA). Electrospray ionization mass spectra (ESI-MS) were recorded using a Thermo Fisher Exactive Orbitrap (Waltham, MA, USA) mass spectrometer coupled to an Advion TriVersa Nanomate (Ithaca, NY, USA) injection system with samples prepared in 100% HPLC-grade CH_3_CN prior to analysis. UV-visible spectrometry studies were carried out on a Shimadzu UV-2600. FT-IR spectra were conducted using a Perkin Elmer Spectrum Two Spectrometer (Waltham, MA, USA). For biological studies, complexes were prepared in molecular-biology-grade DMF and further diluted in 80 mM HEPES buffer (Waltham, MA, USA). DNA plasmids and enzymes were purchased from New England BioLabs (NEB, Ipswich, MA, USA). Solutions of the title complex (Cu-DPQ) for biological studies were carried out in DMF with further dilutions made in 80 mM HEPES.

### 2.2. Synthesis of [Cu(DPQ)_2_NO_3_](NO_3_)

[Cu(DPQ)_2_NO_3_](NO_3_) was prepared by treating 2 equivalents of DPQ (0.0451g, 0.194 mmol) with 1 equivalent of Cu(NO_3_)_2_.3H_2_O (0.0234 g, 0.097 mmol) under ethanolic reflux for 3 h. A green solid was isolated and washed with ice cold EtOH and dried by desiccation (yield: 0.050 g, 78%). ATR-FTIR (cm^−1^): 596, 643, 724, 820, 1016, 1082, 1128, 1213, 1290, 1370, 1392, 1439, 1491, 1582, 3033, 3040. Elemental analysis calculated for: C_28_H_16_CuN_10_O_6_: C, 51.58; H, 2.47; N, 21.48; Cu, 9.75. %Found: C, 51.09; H, 2.28; N, 21.13; Cu, 9.95. ESI-MS: *m*/*z* calculated: 263.5 [M]^2+^ found: 263.6. Solubility: DMSO, DMF.

### 2.3. X-Ray Crystallography

The data were collected at 150(2) K on a Bruker-Nonius Apex II CCD diffractometer using Mo*K*_α_ radiation (λ = 0.71073Å) and were corrected for Lorentz and polarisation effects. Data were processed as a 2-component twin and corrected for absorption. The structure was solved by dual-space methods (SHELXT) [37] and refined on F^2^ using all the reflections (SHELXL) [38]. All the non-hydrogen atoms were refined using anisotropic atomic displacement parameters and hydrogen atoms bonded to carbon were inserted at calculated positions using a riding model, hydrogen atoms of solvate water were not located or included in the refinement since partial-occupancy and disorder meant no single H-bonding network could be identified. Details for data collection and refinement are summarised in Supplementary S2. CCDC 1959974 contains the supplementary crystallographic data for this paper. These data can be obtained free of charge from The Cambridge Crystallographic Data Centre via www.ccdc.cam.ac.uk/data_request/cif.

Crystal Data for [Cu(DPQ)_2_NO_3_](NO_3_)·2H_2_O, C_28_H_20_N_10_O_8_Cu (*M* = 688.08 g/mol): monoclinic, space group *P*2_1_ (no. 4), *a* = 7.3882(12) Å, *b* = 29.205(5) Å, *c* = 13.363(2) Å, *β* = 103.686(3)°, *V* = 2801.4(8) Å^3^, *Z* = 4, *T* = 150 (2) K, *μ*(Mo*K*α) = 0.852 mm^−1^, *Dcalc* = 1.631 g/cm^3^, 5048 reflections measured (4.198° ≤ 2θ ≤ 50.054°), 5048 unique, which were used in all calculations. *R*_int_ = 0.044 (for HKLF 4 data containing 39,211 reflections, with I > 3sig(I), R_sigma_ = 0.0677 The final *R*1 was 0.0566 (*I* > 2σ(*I*)) and *wR*2 was 0.1379 (all data).

### 2.4. DNA Binding Studies

EtBr displacement, viscosity, and topoisomerase-I-mediated DNA relaxation assay were conducted according to methods previously reported by Kellett et al. [39,40]

### 2.5. DNA Damage Studies

Oxidative DNA damage studies including covalent recognition elements and free radical scavengers on pUC19 were carried out according to published methods [33].

## 3. CpG Sequence Studies

### 3.1. PCR Primer Design

Forward and reverse primers for the pBR322 vector (4361 bp) were designed in order to generate a 798 bp sequence (54% GC) by PCR. The transcript, which contains four individual CCGG sequences (CpG islands), was produced using PCR (35 cycles) with 1 ng pBR322 plasmid (NEB, N3033L) using 2× MyTaq Red Mix (Bioline) at suitable annealing temperatures for the primer pair. The sequence length was verified using a 1 Kb DNA ladder. Further details can be found in Supplementary S5.

### 3.2. Restriction Enzyme Studies

The 798 bp amplicon was exposed to a range of enzymes including HpaII (NEB, R0171S), MspI (NEB, R0106S) and HpaII-MT (NEB, M0214S). A quantity of 400 ng of the amplified sequence was treated with each enzyme for 1 h at 37 °C as per the manufactures’ guidelines. Endonuclease recognition at each 5′-C⤋CGG-3′ site was confirmed by agarose gel electrophoresis with HpaII and MspI (isoschizomers). Successful methylation of internal cytosine residues (5mC) of each CCGG island by HpaII methyltransferase was confirmed when HpaII was observed to have no restriction effect due to its sensitivity to CpG methylation. Electrophoresis experiments were performed at 60 V for 60 min on a 2% agarose gel.

### 3.3. DNA Damage

A quantity of 400 ng of the 798 bp amplicon was treated with increasing concentrations of Cu-DPQ in the absence and presence of 1 mM of exogenous reductant (Na-*L*-ascorbate). A further experiment was carried out involving pre-treatment with HpaII methyltransferase and subsequent exposure of the complex in the absence and presence of 1 mM reductant.

## 4. Results and Discussion

### 4.1. Preparation and Characterisation of Cu-DPQ

The dipyrido[3,2-*f*:2′,3′-*h*]quinoxaline ligand (DPQ) was prepared using a previously reported method involving Schiff-base condensation of 1,10-phenanthroline-5,6-dione with ethylenediamine [32]. [Cu(DPQ)_2_(NO_3_)](NO_3_) was generated by treating 2 equivalents of DPQ with 1 equivalent of Cu(NO_3_)_2_.3H_2_O under ethanolic reflux and was isolated by vacuum filtration as a green solid. The complex was characterised by elemental analysis, ESI-MS and attenuated total reflectance (ATR) Fourier transform infrared (FTIR) spectroscopies. [Cu(DPQ)_2_(NO_3_)](NO_3_) was isolated in good yield with elemental analysis results for carbon, hydrogen, nitrogen, and copper, in agreement with theoretical values. ESI-MS analysis contained the expected parent ion peak for the cationic complex [Cu(DPQ)_2_]^2+^ (lane1). Characteristic bands for C=C, C=N and C-N stretching of the DPQ ligand were monitored and shifts in both C=N and C-N were noted when the complex was formed (Figure S2). The electronic spectrum of the complex was measured in the UV-visible range, monitored over time and compared to Cu-Phen and Cu-DPQ-Phen. No notable differences were observed in the *d–d* transition region (600–900 cm^−1^) upon prolonged incubation; however, the appearance of a new band ~445 cm^−1^ in the spectrum of all three complexes indicates potentially significant rearrangement in DMF solution (Figure S3).

**Figure 2:**
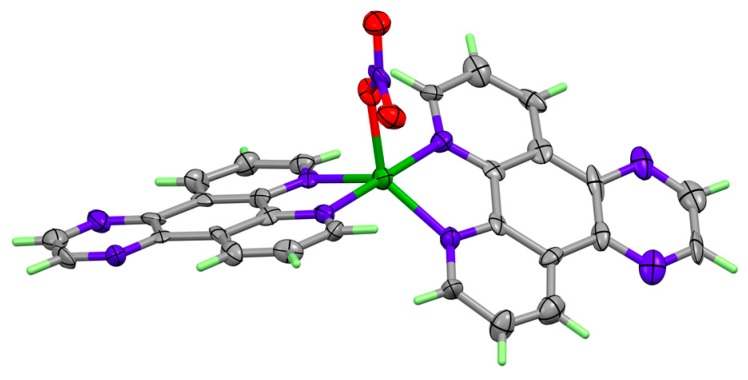
X-ray structure of one of the two independent [Cu(DPQ)_2_NO_3_]^+^ cations showing 50% probability ellipsoids (colour scheme: copper, green; nitrogen, purple; carbon, grey; and oxygen, red).

Single crystal X-ray structure analysis of the dihydrated complex [Cu(DPQ)_2_(NO_3_)](NO_3_)·2H_2_O revealed that the asymmetric unit contains two independent, but similar, [Cu(DPQ)_2_(NO_3_)]^+^ cations. The copper ions are 5-coordinate and their geometry is close to trigonal bipyrimidal with the nitrate anions in the trigonal plane (Figure 2); the τ values [41] are 0.75 and 0.77 for Cu1 and Cu2, respectively. The mean planes of the DPQ ligands are inclined at 46.35(9)° about Cu1 and 43.37(9)° at Cu2. The cations are π-stacked into zig-zag sheets perpendicular to the *c* axis (Figure S4), the spaces between sheets are occupied by the uncoordinated nitrate counter anions and solvate water molecules, which make up a hydrogen-bonded network (incorporating some disorder of both anion and water). The Cambridge Structural Database [42] includes several other examples of structures containing the [Cu(DPQ)_2_X]^n+^ or [Cu(Phen)_2_X]^n+^ cations (X = water or anionic ligand, n = 1 or 2). The structures of these cations are broadly similar to that reported here provided the ligand X is a weak donor [43,44] or if there is no fifth donor [45]. However, if X is thiocyanate, azide or, chloride square planar or tetragonal geometries were observed [46].

### 4.2. DNA Binding Experiments

**Figure 3:**
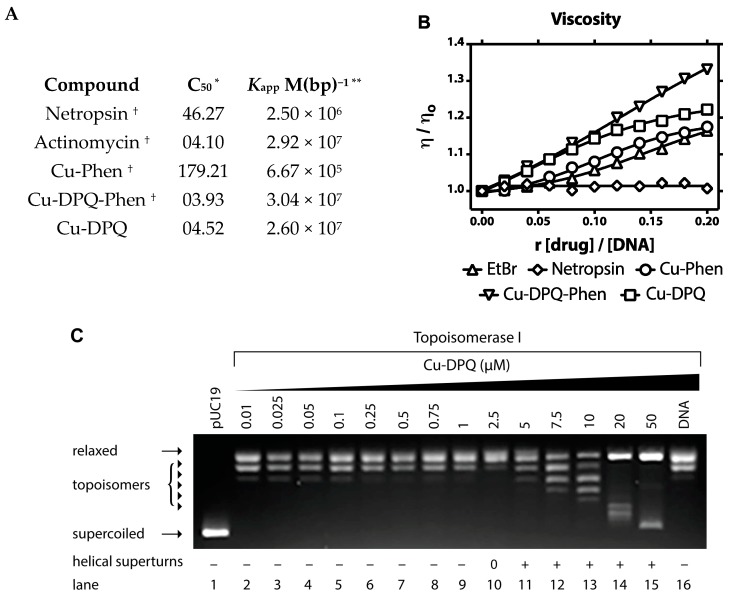
(**A**) Apparent DNA binding constants (*K*_app_) of Cu-DPQ to EtBr-saturated solutions of dsDNA (* C_50_ = concentration required to reduce fluorescence by 50%; ** *K*_app_ = *K*_e_ × 12.6/C_50_ where *K*_e_ = 9.5 × 10^6^ M(bp)^−1^; ^†^ previously reported). (**B**) Relative changes in viscosity profiles of stDNA in the presence of Cu-DPQ, Cu-DPQ-Phen, Cu-Phen, netropsin and EtBr-treated salmon testes dsDNA. (**C**) Topoisomerase-I-mediated DNA relaxation assay.

To establish how Cu-DPQ interacts with duplex DNA a range of binding studies were conducted. Firstly, the apparent binding constant (*K*_app_) of the complex to calf thymus DNA was determined using a high-throughput 96-well plate ethidium bromide (EtBr) displacement assay [39]. DNA was treated with an excess of EtBr, which becomes highly fluorescent when bound to DNA. Solutions were then treated with increasing concentrations of Cu-DPQ in order to reduce the fluorescence intensity, which is indicative of the ejection of bound EtBr from DNA. Cu-DPQ displayed high binding affinity in the range of ~10^7^ M(bp)^−1^. This binding value is superior to that of Cu-Phen (~10^5^ M(bp)^−1^) and broadly in line with high-affinity complexes Cu-DPQ-Phen, Cu-DPPZ-Phen and actinomycin D (Figure 3A) [32]. To probe the DNA binding mode of Cu-DPQ further, viscosity analysis with salmon testes dsDNA fibres was investigated. Here, hydrodynamic changes revealed an increasing trend in viscosity (relative to complex loading) indicative of extension and unwinding effects associated with intercalation. The extent of unwinding elicited by Cu-DPQ surpassed that of Cu-Phen and EtBr (Figure 3B).

The topoisomerase-I-mediated DNA relaxation assay was next applied to study the intercalative effects of Cu-DPQ on supercoiled (SC) plasmid DNA (Figure 3C). This experiment was monitored by gel electrophoresis as DNA isoforms migrate at different rates through agarose due to their size and charge. Generally, intercalation results in the gradual relaxation of negatively (-) supercoiled plasmid to relaxed open circular (0) plasmid and upon further drug loading, the plasmid becomes unwound in the opposite direction resulting in positively (+) coiled SC DNA [47]. Cu-DPQ was observed to relax (-) SC plasmid to its OC form at ~2.5 µM, beyond which (+) SC topoisomers were identified. At high complex concentrations of 20 and 50 µM (lanes 14 and 15), DNA nicking was identified whereby a fixed band remains in line with the OC isoform. These results are broadly equivalent to those observed for the ternary Cu-DPQ-Phen complex (Figure S6A) and represent an ∼8-fold enhancement in the unwinding effects of the semi-intercalator Cu-Phen (Figure S6B).

### 4.3. DNA Damage Studies on SC pUC19

**Figure 4:**
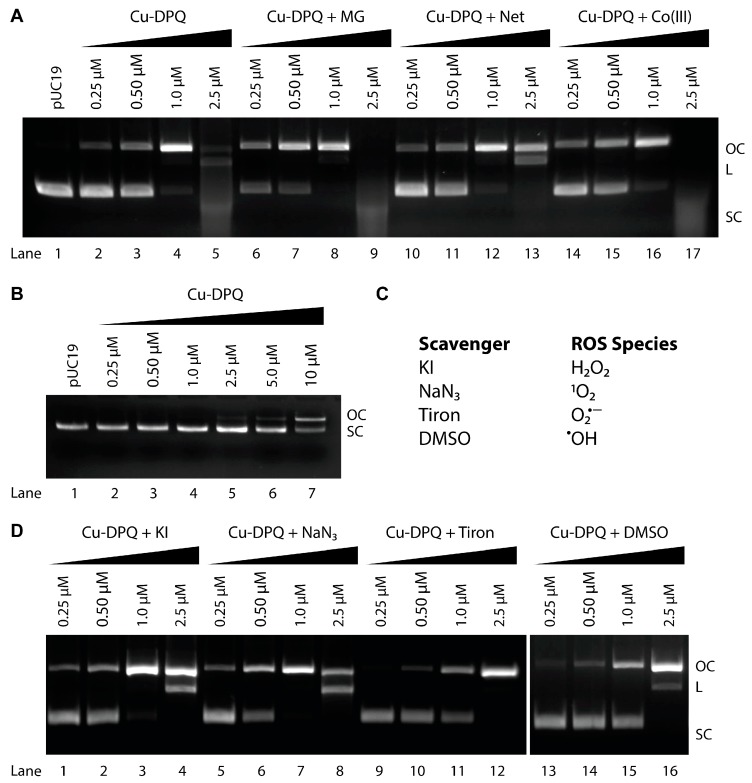
(**A**) DNA cleavage reactions on pUC19 in the presence of non-covalent recognition elements methyl green (MG), netropsin (Net) and cobalt(III) hexamine chloride (Co(III)). (**B**) Nuclease in the absence of reductant. (**C**) ROS scavengers employed in this study. (**D**) DNA cleavage reactions in the presence of selected ROS scavengers.

The cleavage efficiency of Cu-DPQ was monitored by agarose gel electrophoresis over a narrow concentration range of 0.25 µM, 0.50 µM, 1.0 µM, and 2.5 µM (Figure 4A, lanes 2–5). Cu-DPQ was initially reduced to the active Cu(I) state with 1 mM exogenous reductant (Na-*L*-ascorbate) prior to the introduction of plasmid DNA. Cu-DPQ showed concentration-dependent relaxation of SC to OC with almost complete degradation upon treatment with 2.5 µM of the complex. The cleavage efficiency of Cu-DPQ on pUC19 is greater than Cu-DPQ-Phen and Cu-Phen complexes, illustrating the influence of the bis-ligated DPQ ligand on oxidative DNA damage [33]. Interestingly, in the absence of reductant, Cu-DPQ is capable of ‘self-activation’ since the emergence of OC DNA is visible in the presence of 2.5 µM of this complex with higher concentrations emerging at 10 µM (Figure 4B, lane 7).

To determine how the complex cleaves plasmid DNA, pUC19 was pre-treated with an excess of non-covalent recognition agents including methyl green (major groove binder), netropsin (minor groove binder) and cobalt(III) hexamine chloride (electrostatic binder). Pre-incubation with methyl green (Figure 4A lanes 6–9) resulted in enhanced nuclease activity compared to methyl green free experiments (lanes 2–5). However, priming the plasmid with netropsin (lanes 10–13) afforded notable protection to the minor groove resulting in a reduction in oxidative DNA damage. Finally, pre-treatment with the electrostatic binding agent had negligible effects (lanes 14–17). These results are in agreement with those previously reported with Cu-Phen, Cu-Phen-DPQ and Cu-TPMA-N,N′ complexes where the presence of methyl green results in enhanced chemical nuclease activity due to its minor groove priming effects [1,33,34].

To probe the DNA damage profile of Cu-DPQ, a range of radical scavengers were introduced to the nuclease experiment to investigate the nature of ROS species involved in DNA oxidation (Figure 4C). This study identified the superoxide radical (O_2_^•−^) as a key radical involved in the scission process as pre-treatment with tiron (4,5-dihydroxy-1,3-benzenedisulfonic acid disodium salt) significantly impeded oxidative damage to the plasmid (Figure 4D, lane 12). Moderate protection was afforded by potassium iodide (KI, Figure 4D, lanes 1–4) and dimethyl sulfoxide (DMSO, Figure 4D, lanes 13–16), which scavenge H_2_O_2_ and ^•^OH species, respectively. Singlet oxygen (^1^O_2_), however, does not appear to play a significant role in the oxidative cleavage mechanism of Cu-DPQ. The overall trend in damage inhibition follows O_2_^•−^ > H_2_O_2_ ≃ ^•^OH > ^1^O_2_ and departs substantially from Cu-Phen and Cu-DPQ-Phen where hydroxyl radical generation predominates [33]. Interestingly, we previously identified that O_2_^•−^ plays a major role in oxidative profile of [Cu(o-phthalate)(1,10-phenanthroline)] and the Cu-TPMA-N,N′ series [31,34]. Finally, copper chelating agents ethylenediaminetetraacetic acid (EDTA) and neocuprione were shown to afford full protection to the SC substrate (Figure S7).

## 5. Studies with CpG and Methylated CpG Islands

**Figure 5:**
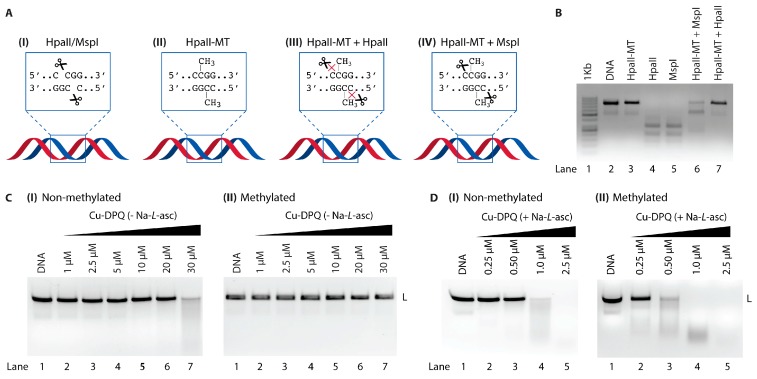
(**A**) Cartoon representation of enzyme restriction sites. (**B**) Control experiment with isoschizomers HpaII and MspI in the presence and absence of HpaII-MT. (**C**) A quantity of 400 ng of 798 bp linear sequence (**I** non-methylated and **II** methylated) treated with Cu-DPQ in the absence of reductant. (**D**) 400 ng of 798 bp linear sequence (**I** non-methylated and **II** methylated) treated with Cu-DPQ in the presence of reductant Na-*L*-asc.

To probe the DNA oxidation profile of Cu-DPQ further, cleavage experiments involving a 798 bp dsDNA sequence (amplified from pBR322) containing four distinct cytosine–phosphate–guanine (CpG) islands (5′-CCGG-3′) were undertaken. The internal cytosine residues of this transcript were methylated using HpaII methyltransferase (HpaII-MT) to generate an equivalent amplicon with four 5′-C5mCGG-3′ sites (Figure 5AII and Figure 5B lane 3). To verify the presence of four CpG islands, the non-methylated sequence was treated with HpaII and isoschizomer MspI. Both enzymes recognise and cleave the 5′-C⤋CGG-3′ tract to produce the desired fragmentation pattern (Figure 5AI and Figure 5B lanes 4 and 5; supplementary S5). HpaII is sensitive to CpG methylation and was inactive against the 5mC sequence (Figure 5AIII and Figure 5B lane 7), while isoschizomer MspI is insensitive to DNA methylation and cleaved all four methylation sites (Figure 5AIV and Figure 5B lane 6).

Cleavage experiments with Cu-DPQ in the absence of reductant were then examined using non-methylated and methylated sequences (Figure 5CI and II). At the highest tested concentration (30 µM), the non-methylated sequence was extensively sheared while the 5mC amplicon remained intact. Similar experiments were then conducted in the presence of reductant (Figure 5DI and II). Here, the activated Cu-DPQ complex degraded the non-methylated sequence at 1.0 µM, while shearing of the methylated transcript started at 0.25 µM with almost complete ablation at 0.5 µM. These results indicate that under ‘self-activation’ conditions (i.e., without reductant), DNA cleavage may occur in the major groove of CpG islands. When these islands contain 5mC residues, the methyl group serves to sterically block the major groove, thereby preventing Cu-DPQ-mediated oxidation. In its reduced Cu(I) form, Cu-DPQ appears to be a potent oxidant of the minor groove since limiting access to the major groove of CpG islands enhances oxidation. Therefore, 5mC serves to direct the complex to the minor groove—in much the same way as methyl green—and this residency serves to increase the oxidative cleavage effects of Cu-DPQ in its reduced form.

## 6. Conclusions

The Cu-DPQ complex was generated in high purity and its structure determined by single-crystal X-ray crystallography. In the solid state, the crystal of [Cu(DPQ)_2_(NO_3_)](NO_3_)·2H_2_O revealed the asymmetric unit contains two independent, but similar, [Cu(DPQ)_2_(NO_3_)]^+^ cations. The five-coordinated copper(II) ions [CuN_4_O] adopt a geometry close to trigonal bipyrimidal with the nitrate anions in the trigonal plane. Cu-DPQ is a high-affinity DNA binder with potent intercalative properties compared to Cu-Phen, as evidenced by the DNA fluorescence displacement assay, viscosity and topoisomerase-I DNA unwinding studies. The complex is capable of ‘self-activation’ by inducing DNA damage in the absence of exogenous reductant but is greatly enhanced in its presence. The oxidative DNA damage profile of Cu-DPQ was studied in the presence of free radical scavenging species, with results demonstrating the complex to be an efficient oxidiser of pUC19 plasmid DNA mediating oxidative DNA damage predominately through the generation of the superoxide radical species (O_2_^•−^) with involvement from the hydroxyl radical (^•^OH). By pre-exposing plasmid DNA with non-covalent steric blocking agents of methyl green (major groove) or netropsin (minor groove), the minor groove was identified as the preferred DNA oxidation site. To help confirm this effect, we introduced 5-methylcytosine (5mC) into a 798 bp construct at four individual CpG islands. Here, 5mC served to sterically block the major groove and enhance chemical nuclease activity, thereby supporting preferential oxidative cleavage at the minor groove. This mono-nuclear copper(II) DNA damaging agent represents an interesting therapeutic lead for the treatment of human cancer and warrants future in vitro evaluation.

## Supplementary Materials

Supplementary materials are available online.
