# Supplementary material for: Copper bis-Dipyridoquinoxaline Is a Potent DNA Intercalator that Induces Superoxide-Mediated Cleavage via the Minor Groove
Source: Molecules. 2019 Nov 26;24(23):4301. doi: 10.3390/molecules24234301 (PMC6930674; doi:10.3390/molecules24234301)
Supplement: Supplementary file 1 [file molecules-24-04301-s001.pdf]

Copper *bis*-dipyridoquinoxaline is a potent DNA intercalator that induces superoxide-mediated cleavage *via* the minor groove

Zara Molphy,[1,2] Vickie McKee,[1,3] and Andrew Kellett[1,2]

[1] *School of Chemical Sciences, National Institute for Cellular Biotechnology and Nano Research Facility, Dublin City University, Glasnevin, Dublin 9, Ireland.*
[2] *Synthesis and Solid-State Pharmaceutical Centre, School of Chemical Sciences, Dublin City University, Glasnevin, Dublin 9, Ireland.*
[3] *Department of Physics, Chemistry and Pharmacy, University of Southern Denmark, Campusvej 55, 5230 Odense M, Denmark.*

(S1) Characterisation of Cu-DPQ
(S2) X-Ray Crystallography
(S3) DNA binding experiments
(S4) DNA damage studies on SC pUC19
(S5) DNA damage studies on linear DNA

(S1) Characterisation of $[Cu(DPQ)_2(NO_3)](NO_3)$

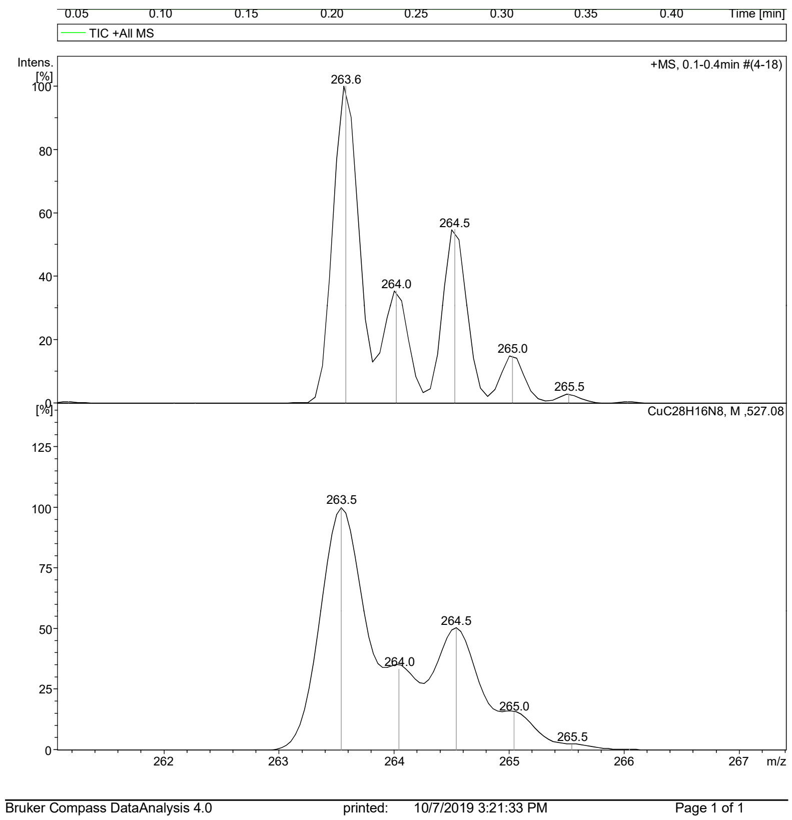

Figure. S1 ESI-MS spectra of Cu-DPQ complex.

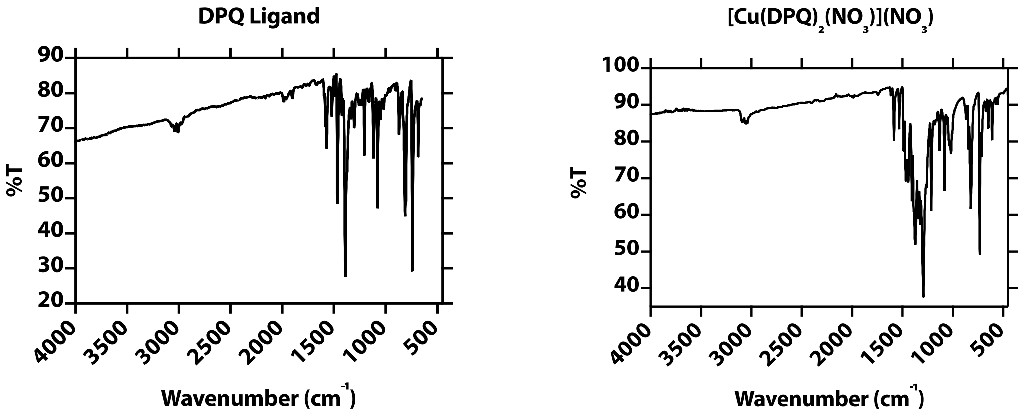

Figure. S2 ATR-FTIR spectra of DPQ ligand $[Cu(DPQ)_2(NO_3)](NO_3)$.

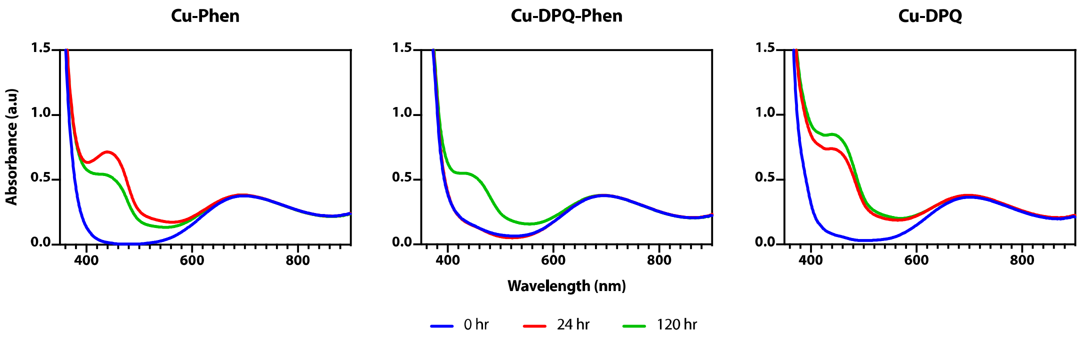

Figure. S3 UV-Vis stability study of Cu-Phen, Cu-DPQ-Phen and Cu-DPQ (5 mM DMF).

(S2) X-ray crystallography of $[Cu(DPQ)_2(NO_3)]NO_3{\cdot}2H_2O$

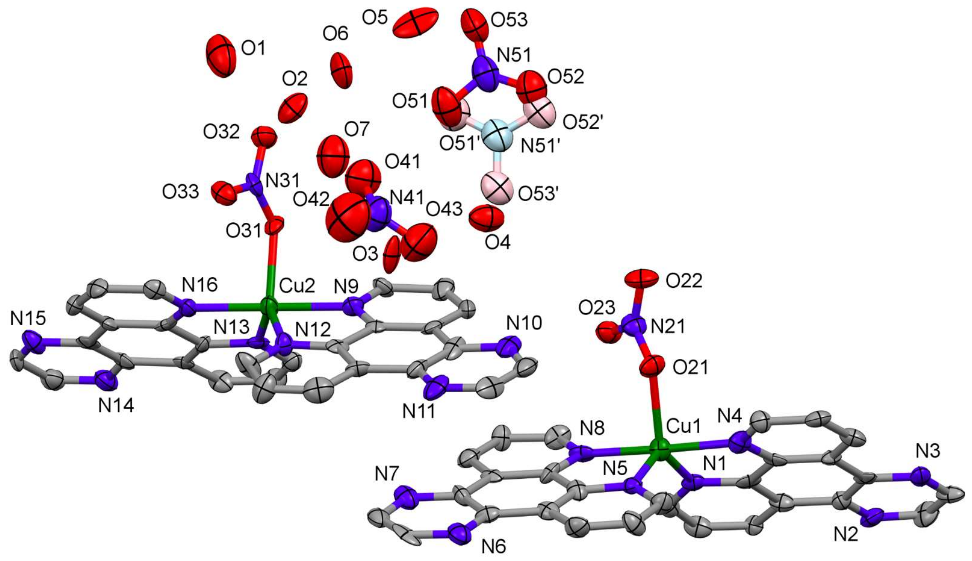

Figure S4. Structure of $[Cu(DPQ)_2(NO_3)](NO_3){\cdot}2H_2O$ showing the two independent cations and the labelling scheme for the non-carbon atoms. Hydrogen atoms omitted for clarity, 50% probability ellipsoids.

The data were collected at 150(2)K on a Bruker-Nonius Apex II CCD diffractometer using MoK_α radiation (λ = 0.71073Å) and were corrected for Lorentz-polarisation effects. Data were processed as a 2-component twin (by rotation about reciprocal axis [-0.002 0.001 1.000]) and corrected for absorption (TWINABS). The structure was solved by dual space methods (SHELXT) and refined on F^2 in SHELXLE using all the reflections (SHELXL with HKLF 5 data). All the non-hydrogen atoms were refined using anisotropic atomic displacement parameters and hydrogen atoms bonded to carbon were inserted at calculated positions using a riding model. Hydrogen atoms of solvate water were not located or included in the refinement since partial-occupancy and disorder meant no single H-bonding network could be identified (Fig 3). One of the two uncoordinated nitrate anions was disordered and modelled with 60:40 occupancy of two overlapping sites. Only one of the solvate water molecules (O1) was full occupancy, the rest were refined with 50% occupancy over six independent sites. Parameters for data collection and refinement are summarised in Table 1. $[Cu(DPQ)_2(NO_3)](NO_3){\cdot}2H_2O$ is a solvomorph of previously reported structure $[Cu(DPQ)_2(NO_3)](NO_3)$.[1]

Table S1 Crystal data for $[Cu(DPQ)_2(NO_3)](NO_3)\cdot 2H_2O$

$C_{28}H_{16}CuN_9O_3\cdot NO_3\cdot 2(H_2O)$	$F(000) = 1404$
$M_r = 688.08$	$D_x = 1.631$ Mg m^{-3}
Monoclinic, $P2_1$	Mo $K\alpha$ radiation, $\lambda = 0.71073$ Å
$a = 7.3882$ (12) Å	Cell parameters from 4719 reflections
$b = 29.205$ (5) Å	$\theta = 2.6$–$22.3°$
$c = 13.363$ (2) Å	$\mu = 0.85$ mm^{-1}
$\beta = 103.686$ (3)°	$T = 150$ K
$V = 2801.4$ (8) Å^3	Lath, pale green
$Z = 4$	0.64 × 0.12 × 0.02 mm

Data collection

Bruker APEX 2 CCD diffractometer	5048 independent reflections
Graphite monochromator	4386 reflections with $I > 2\sigma(I)$
ω rotation with narrow frames scans	$\theta_{max} = 25.0°$, $\theta_{min} = 2.1°$
Absorption correction: multi-scan TWINABS-2012/1 (Bruker,2012) was used for absorption correction. Final HKLF 4 output contains 39211 reflections, Rint = 0.0845 (12298 with I > 3sig(I), Rint = 0.0444)	$h = -8\rightarrow 8$
$T_{min} = 0.579$, $T_{max} = 0.745$	$k = 0\rightarrow 34$
5048 measured reflections	$l = 0\rightarrow 15$

Refinement

Refinement on F^2	Secondary atom site location: difference Fourier map
Least-squares matrix: full	Hydrogen site location: inferred from neighbouring sites
$R[F^2 > 2\sigma(F^2)] = 0.057$	H-atom parameters constrained
$wR(F^2) = 0.138$	$w = 1/[\sigma^2(F_o^2) + (0.0874P)^2 + 0.8348P]$ where $P = (F_o^2 + 2F_c^2)/3$
$S = 1.07$	$(\Delta/\sigma)_{max} < 0.001$
5048 reflections	$\Delta\rangle_{max} = 0.64$ e Å^{-3}
911 parameters	$\Delta\rangle_{min} = -0.68$ e Å^{-3}
333 restraints	Absolute structure: No quotients, so Flack parameter determined by classical intensity fit
Primary atom site location: dual	Absolute structure parameter: 0.08 (2)

Cell refinement: *SAINT* v8.34A (Bruker, 2013); data reduction: *SAINT* v8.34A (Bruker, 2013); program(s) used to solve structure: SHELXT[2]; program(s) used to refine structure: *SHELXL2018*/3[3] and shelXle[4]; molecular graphics: *Mercury*[5]; software used to prepare material for publication: Olex2[6], *publCIF*.[7]

Table S2. Selected Geometric parameters (Å, °) for $[Cu(DPQ)_2(NO_3)](NO_3)\cdot 2H_2O$

Cu1—N1	2.041 (11)	Cu2—N9	2.035 (11)
Cu1—N4	2.006 (10)	Cu2—N12	2.052 (10)
Cu1—N5	2.089 (9)	Cu2—N13	2.089 (11)
Cu1—N8	1.949 (11)	Cu2—N16	1.979 (10)
Cu1—O21	2.144 (9)	Cu2—O31	2.092 (8)

Table S3. Potential hydrogen bond distances (Å) for $[Cu(DPQ)_2(NO_3)](NO_3)\cdot 2H_2O$

O1 - O2	2.75 (0.02)
O1 - O21_$1	2.92 (0.01)
O2 - O6	2.74 (0.04)
O2 - O41	3.04 (0.03)
O2 - O42	2.98 (0.03)
O3 - O4	2.57 (0.03)
O3 - O6_$2	2.77 (0.03)
O3 - O7	3.09 (0.03)
O3 - O42	2.87 (0.03)
O4 - O51	2.90 (0.04)
O4 - O53_$2	3.00 (0.03)
O5 - O43_$3	2.77 (0.03)
O5 - O53	2.71 (0.03)
O6 - O51	2.77 (0.03)
O6 - O51'	2.94 (0.04)
O6 - O7	2.63 (0.04)
O7 - O32	3.04 (0.03)
O7 - O51	3.17 (0.03)

Symmetry equivalents: $1 -1-x, -1/2+y, 1-z, $2 1+x, y, z, $3 -1+x, y, z

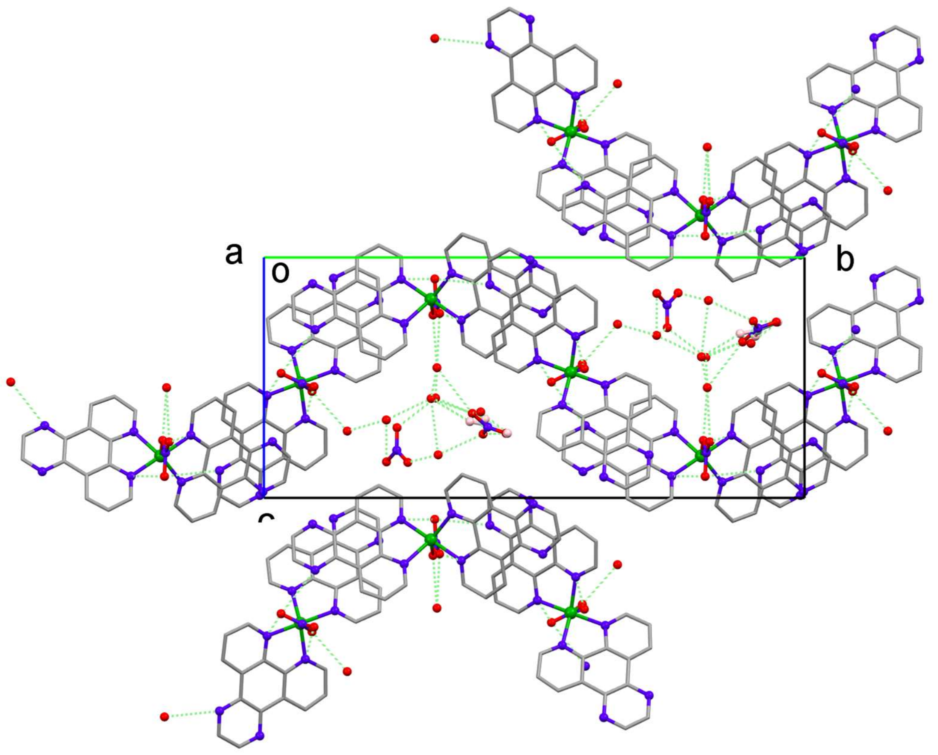

Figure S5. Unit cell plot for $[Cu(DPQ)_2(NO_3)](NO_3)\cdot 2H_2O$ showing H-bonding (dashed green lines) and face stacking of the DPQ ligands.

(S3) DNA binding experiments

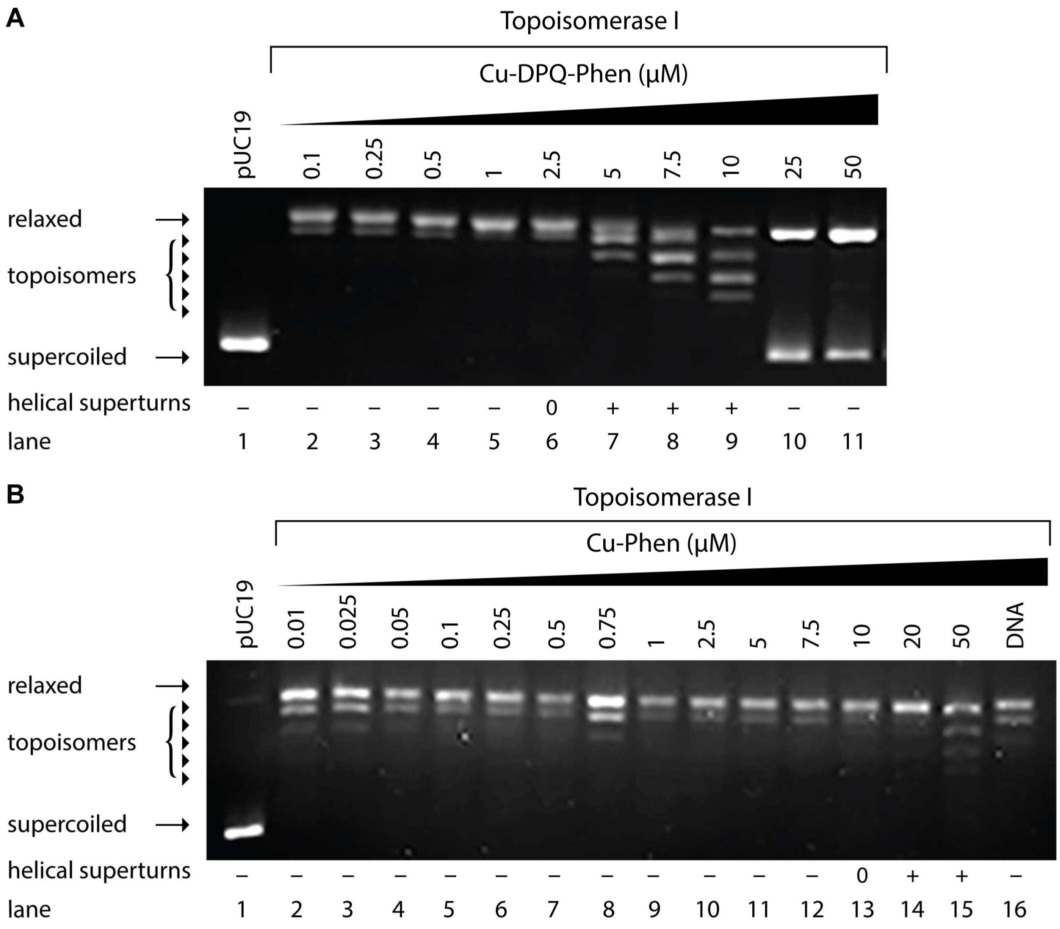

Figure S6. Topoisomerase-I mediated DNA relaxation assay in the presence of A. Cu-Phen and B. Cu-DPQ-Phen.

(S4) DNA damage studies on SC pUC19

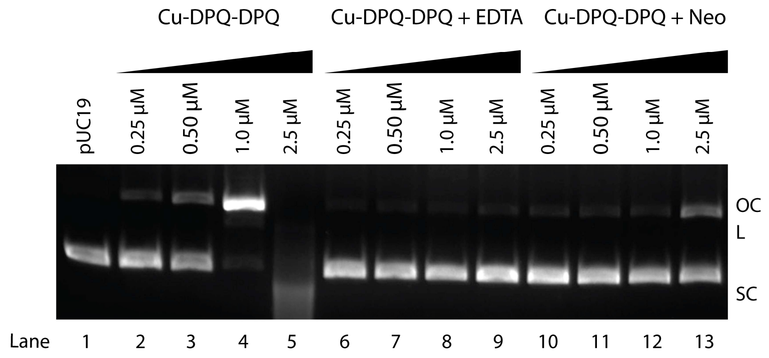

Figure S7. DNA cleavage reactions of Cu-DPQ-DPQ in the presence of 1 mM Na-*L*-Asc on pUC19 (lanes 2-5). Pre-treatment of plasmid with 100 µM EDTA (lanes 6-9) or 100 µM neocuprione (lanes (10-13) prior to complex exposure.

(S5) DNA damage studies on linear DNA

Sequence generated by PCR:

Gctgcaaaacgtctgcgacctgagcaacaacatgaatggtcttcggtttccgtgtttcgtaa
agtctggaaacgcggaagtcagcgccctgcaccattatgtt**ccgg**atctgcatcgcaggatg
ctgctggctaccctgtggaacacctacatctgtattaacgaagcgctggcattgaccctgag
tgatttttctctggtcccgccgcatccataccgccagttgtttaccctcacaacgttccagt
aa**ccgg**gcatgttcatcatcagtaacccgtatcgtgagcatcctctctcgtttcatcggtat
cattacccccatgaacagaaatcccccttacacggaggcatcagtgaccaaacaggaaaaaa
ccgcccttaacatggcccgctttatcagaagccagacattaacgcttctggagaaactcaac
gagctggacgcggatgaacaggcagacatctgtgaatcgcttcacgaccacgctgatgagct
ttaccgcagctgcctcgcgcgtttcggtgatgacggtgaaaacctctgacacatgcagctc**c**
cggagacggtcacagcttgtctgtaagcggatg**ccgg**gagcagacaagcccgtcagggcgcg
tcagcgggtgttggcgggtgtcggggcgcagccatgacccagtcacgtagcgatagcggagt
gtatactggcttaactatgcggcatcagagcagattgtactgagagtgcaccatatgcggtg
tgaaataccgcacagatgcgtaaggagaaaataccgcatcaggcgctcttccgc

Forward: 5′-gctgcaaaacgtctgcgacc-3′,
Reverse: 5′-cgcatcaggcgctcttccgc-3′

Treatment with HpaII and MspI results in 5 bands of varying length:

1. Start → first internal CCGG – 104 bases
2. 1st CCGG → 2nd CCGG – 147 bases
3. 2nd CCGG → 3rd CCGG – 307 bases
4. 3rd CCGG → 4th CCGG – 34 bases
5. 4th CCGG → end – 206 bases

References

1 D. İnci, R. Aydın, H. Huriyet, Y. Zorlu and N. Çinkılıç, *Applied Organometallic Chemistry*, 2018, **32**, e4309.
2 G. M. Sheldrick, *Acta Crystallography*, 2015, **A71**, 3–8.
3 G. M. Sheldrick, *Acta Crystallography*, 2015, **C71**, 3–8.
4 C. B. Hübschle, G. M. Sheldrick and B. Dittrich, *J. Appl. Crystallogr.*, 2011, **44**, 1281–1284.
5 C. F. Macrae, I. J. Bruno, J. A. Chisholm, P. R. Edgington, P. McCabe, E. Pidcock, L. Rodriguez-Monge, R. Taylor, J. van de Streek and P. A. Wood, *J Appl Cryst*, 2008, **41**, 466–470.
6 O. V. Dolomanov, L. J. Bourhis, R. J. Gildea, J. a. K. Howard and H. Puschmann, *J Appl Cryst*, 2009, **42**, 339–341.
7 S. P. Westrip, *J. Appl. Crystallogr.*, 2010, **43**, 920–925.
